# Deprescribing medications for older adults in the primary care context: A mixed studies review

**DOI:** 10.1002/hsr2.45

**Published:** 2018-05-10

**Authors:** Robyn J. Gillespie, Lindsey Harrison, Judy Mullan

**Affiliations:** ^1^ School of Medicine, Faculty of Science, Medicine and Health University of Wollongong Wollongong Australia; ^2^ School of Health and Society, Faculty of Social Sciences University of Wollongong Wollongong Australia; ^3^ Centre for Health Research Illawarra Shoalhaven Population (CHRISP), Australian Health Services Research Institute University of Wollongong NSW 2522 Australia

**Keywords:** deprescribing, general practice, mixed studies review, older adults, polypharmacy, primary care

## Abstract

**Aims:**

This review investigates the factors that influence deprescribing of medications in primary care from the perspective of general practitioners (GPs) and community‐living older adults.

**Methods:**

A mixed studies review structure was adopted searching Scopus, CINAHL, PsychINFO, ProQuest, and PubMed from January 2000 to December 2017. A manual search of reference lists was also conducted. Studies were included if they were original research available in English and explored general deprescribing rather than deprescribing of a specific class of medications. The Mixed Methods Assessment Tool was used to assess the quality of studies, and content analysis generated common categories across studies.

**Results:**

Thirty‐eight articles were included, and 7 key categories were identified. The review found that the factors that influence deprescribing are similar across and within health systems and mostly act as barriers. These factors remained unchanged across the review period. The structural organisation of health systems remains poorly suited to facilitate deprescribing. Individual knowledge gaps of both GPs and older adults influence practices and attitudes towards deprescribing, and significant communication gaps occur between GPs and specialists and between GPs and older adults. As a result, deprescribing decision making is characterised by uncertainty, and deprescribing is often considered only when medication problems have already arisen. Trust plays a complex role, acting as both a barrier and facilitator of deprescribing.

**Conclusions:**

Deprescribing is influenced by many factors. Despite recent interest, little change has occurred. Multilevel strategies aimed at reforming aspects of the health system and managing uncertainty at the practice and individual level, notably reducing knowledge limitations and closing communications gaps, may achieve change.

## 1 INTRODUCTION

Polypharmacy use in older adult populations is increasing.^1^, ^2^ A number of reasons have been suggested for this, including the use of disease specific treatment guidelines in the context of multiple morbidities, lower treatment thresholds for chronic diseases,^3^ the ongoing use of preventives,^4^ and the medicalisation of some of the normal processes of aging.^5^ The use of polypharmacy, commonly identified as taking five or more concurrent medications, may be appropriate in some circumstances.^6^ However, it also places older adults at a potentially higher risk of adverse drug reactions,^7^ resulting in increased personal and health care costs, hospitalisations, poor health outcomes,^8^ and/or increased mortality rates.^9^ This level of risk is considerable^10^ and as such, warrants further attention as a public health^11^ and ethical issue.^12^

Medications are rarely indicated for lifelong usage, as many factors may change during the course of treatment.^13^ This is due, in part, to the complexity of managing multiple morbidities experienced by many older adults, the lack of evidence available to inform decisions and changes in health, frailty, and, often, autonomy.^14^ As a result, older adults and their general practitioners (GPs) face particular challenges when deciding on the most appropriate treatment regimens.

Deprescribing has been suggested as one intervention to reduce inappropriate polypharmacy.^7^ The term deprescribing was first used by Woodward^15^ and is defined as a systematic process supervised by a medical professional to reduce or discontinue long‐term medications.^16^ Deprescribing is indicated where the existing or potential harms outweigh existing or potential benefits of a particular medication/medications. Deprescribing may be relevant at any point in the life course, although it is most often considered in the context of medication use for older adults who, as a group, are growing exponentially worldwide.^17^ Ideally, the process of deprescribing takes into account changes in the context of an individual's treatment goals, their current level of functioning, life expectancy, values, and preferences.^18^ However, it is not a process that is routinely considered in primary care.^7^ This is despite evidence of the usefulness of deprescribing to address polypharmacy and reduce mortality,^19^ and an increased awareness of deprescribing at both international^20^ and at national levels.^6^, ^21^, ^22^

Three previous literature reviews in this research area are available.^23^, ^24^, ^25^ One examined the barriers and facilitators of deprescribing from the perspective of patients, using a narrative synthesis of qualitative, quantitative, and mixed methods studies.^25^ Anderson et al,^23^ on the other hand, sought to understand barriers and facilitators from the perspective of the prescriber, conducting a synthesis of qualitative studies. These initial reviews by Reeve et al^25^ and Anderson et al^23^ were conducted just as interest in this research area gained momentum with most papers being published^26^ since 2015. As such, they were exploratory and broad in scope, being conducted across all health care settings, targeting deprescribing in the context of all adult aged patient groups, and included both general deprescribing and deprescribing of single medication types. A further ethnographic review by Bokhof and Junius‐Walker^24^ had a narrower focus, synthesising findings from qualitative research, to investigate the management of and attitudes towards reducing polypharmacy (including deprescribing), from the perspective of both community‐living older adults and GPs. This is important because, as mentioned earlier, deprescribing is most often considered in the context of the treatment of older adults.

The purpose of the current review is to build on Bokhof and Junius‐Walker^24^ work by investigating the factors that influence deprescribing from the perspective of both GPs and adults aged 65 years or older. Independent, community‐living older adults are the focus of this review, as the majority of this group retain autonomy and are capable of being responsible for their own health decisions,^17^ hence the importance of considering deprescribing from their perspective. Given the increasing evidence that is now available on this topic, a review that incorporates all study types is warranted. Interventions to promote deprescribing will require change on the part of both prescribers and older adults, so it is important to consider how their views interact to create the context where deprescribing discussions can take place.

## 2 METHODS

A mixed studies review methodology was used as a guide to explore the factors that influence deprescribing.^27^, ^28^

### 2.1 Eligibility criteria

#### 2.1.1 Inclusion criteria

Full text primary research articles were included that were available in English, published between January 2000 and December 2017, and that investigated deprescribing or medication cessation/discontinuation in the primary care context for older adults, living independently in the community. General deprescribing was targeted. Articles were also included where deprescribing was mentioned as a mechanism to reduce polypharmacy or potentially inappropriate medications or in the context of the management of multiple morbidities.

#### 2.1.2 Exclusion criteria

Deprescribing of specific medications or medication classes were not included, as unique factors related to individual medication use, such as specific withdrawal issues, might have influenced deprescribing practices and attitudes in these instances. Articles discussing deprescribing within long‐term residential aged care facilities were excluded, as the nature of such care changes the relationship between primary care providers and older adults, limiting the ability of older adults to act autonomously.^29^ Similarly, articles discussing deprescribing within acute care hospital settings were excluded, as it is less likely that there is an established, ongoing relationship between the prescriber and older adult in these settings. Articles were also excluded if they only related to discontinuing medications during palliative stages of care, as the context of medication deprescribing is markedly different in these scenarios.^30^

### 2.2 Literature search

**Figure 1:**
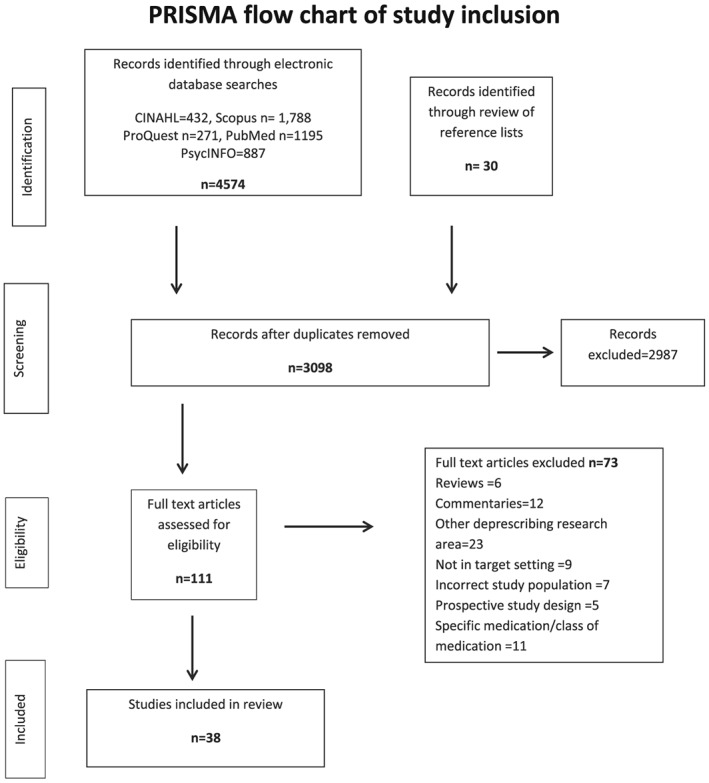
PRISMA flow chart of study inclusion

An initial scoping search was conducted in August 2016; however, due to the rapid proliferation of research in this area, a further search of the following 5 databases was conducted in November 2017: CINAHL, PsychINFO, ProQuest, PubMed, and Scopus. The results are shown in Figure 1.

The search was conducted using combinations of the following search terms and the Boolean operator “AND.”

- “older adult” OR senior* OR elder*
- “general practice” OR “general practitioner” OR GP OR “family physician” OR physician OR “primary care” OR doctor OR clinician OR prescriber OR “health professional” OR “health care professional”
- deprescribe* OR discontin* OR cessation OR cease OR withdraw*OR stop* OR reduc* OR optim*
- polypharmacy OR medication OR medicines OR prescribing OR “prescription drug”

An example search strategy is detailed in Appendix S1.

Search terms were applied to abstracts, keywords, and titles. In addition, a secondary search of reference lists of relevant articles was conducted, to check for other potentially eligible articles. The search was conducted by the main author, following refinement of the search terms under the guidance of a university librarian.

### 2.3 Identifying and selecting relevant articles

After duplicate citations were removed, one reviewer (RG) screened titles for relevant articles. Abstracts and/or full texts were reviewed if the article met the inclusion criteria. Key data from potential articles were extracted into a table. This was reviewed by all authors to confirm the relevance and appropriateness of each article. As the focus of the review was on autonomous community‐living adults, any articles that included community‐living older adults with cognitive impairment were excluded.

### 2.4 Quality assessment of included articles

The quality of the included articles was assessed using the Mixed Methods Appraisal Tool (MMAT).^31^ This checklist is suited for use when studies with a range of methodological techniques need to be assessed. The first 2 authors screened the studies to determine their quality rating, applying the scoring system described in Pluye et al,^32^ and scores were confirmed by the third author.

### 2.5 Analytic approach

The findings sections of included studies were analysed using content analysis.^33^ Findings were explored across all articles to identify factors influencing deprescribing. The first author became familiar with the content by reading each study multiple times. Unique codes were identified and tabled. The relationship between codes was considered in order to organise the codes into groups. These groupings were discussed by all researchers in order to refine the final categories. Seven key categories were generated from the data.

## 3 RESULTS

Searches of electronic databases and the reference lists of related articles identified 4604 potentially relevant articles. Following the application of the exclusion and inclusion criteria, 38 articles remained and were reviewed (Figure 1). Table 1 summarises the data from the included studies. The studies were conducted across a number of countries with varied health systems. Only 2 studies included the perspectives of both GPs and medication users.^61^, ^68^ Thirteen studies included only older adult participants. The remainder included GPs (family physicians) (n = 16) or a mix of primary care prescribers including GPs, nurse practitioners, GPs' assistants, consulting pharmacists, and geriatricians (n = 7).

**Table 1:** Summary of research articles included in the review

Author/Year	Country	Research Aim/s	Sample Size		Data Collection Methods	Data Analysis	MMAT Appraisal Scores, %
			Health Care Providers	Older Adults			
Anderson et al^34^ 2007	Australia	To explore the views of GPs and consultant pharmacists regarding inappropriate polypharmacy and deprescribing in primary care	32 GPs 15 consultant pharmacists (CPs)	N/A	Focus groups	Thematic analysis	100
Anthierens et al^35^ 2010	Belgium	To describe GPs' perspective and beliefs about polypharmacy. Identify the role of the GP in improving prescribing	65 GPs	N/A	Semistructured interviews	Content analysis	75
Bagge et al^36^ 2013	New Zealand	To explore the attitudes of older adults age 75 or older towards their medicines	N/A	60	Semistructured interviews	Thematic analysis	75
Bell et al.^37^ 2015	Norway	To explore the factors that might influence prescribers to deprescribe falls‐risk‐increasing drugs	13 GPs	N/A	Focus groups.	Systematic text condensation	100
Cantrill et al^38^ 2000	UK	To explore factors that influence prescribers in general practice to continue inappropriate long‐term medications	22 GPs	N/A	Semistructured interviews	Thematic analysis	50
Clyne et al^39^ 2013	Ireland	To test the acceptance by GPs of a proposed intervention to reduce potentially inappropriate prescribing in older adults	5 GPs	N/A	Focus group followed by semistructured interviews	Thematic analysis	75
Clyne et al^40^ 2016	Ireland	To explore GP views on prescribing of potentially inappropriate medications in older adults	17 GPs	N/A	Interviews	Thematic analysis	100
Clyne et al^41^ 2017	Ireland	To explore influences and the beliefs and attitudes toward medications of community‐living, older adults' with polypharmacy.	N/A	196	Secondary analysis of cluster randomised control trial data Questionnaire and semi structured interviews	Multiple regression and thematic analysis	75
Elliot et al^42^ 2007	USA	To understand how older adults taking multiple medications make decision.	N/A	20	Semistructured interviews	Constant comparison	75
Farrell et al^43^ 2015	Canada	To identify medication classes where evidence based deprescribing guidelines would be most useful	65 participants (round 1) (8 geriatricians 35 pharmacists 11 GPs 10 nurse practitioners) n = 47 in round 3	N/A	Modified Delphi approach	Content analysis and significance testing	Not scored
Farrell et al^44^ 2018	Canada	To determine if the use of deprescribing guidelines would change prescriber's perception of self‐efficacy and enable deprescribing	50 (38 GPs) participants giving 79 responses across 4 survey rounds	N/A	Questionnaire	Descriptive statistics, longitudinal analysis	25
Fried et al^45^ 2008	USA	To explore what older adults with multimorbidity value when deciding their medication treatment preferences.	N/A	66	Focus groups	Grounded theory approach using constant comparative method	75
Fried et al^46^ 2011	USA	To understand primary care clinicians treatment decision‐making processes for older patients with multiple diseases	40 participants: (36 GPs 2 NP 1 physician assistant 1 pharmacist)	N/A	Focus groups	Content analysis	75
Fried et al^47^ 2016	USA	To develop strategies for identifying and addressing problems with medication regimens for older adults with multiple medical conditions	9 representatives of the disciplines of nursing, medicine and pharmacy n = 8 completed rounds 2 and 3	N/A	Modified Delphi approach	Analysis of Likert scale responses	Not scored
Ie et al^48^ 2017	USA	To investigate variability in prescribing especially of potentially inappropriate prescribing.	61 GPs 2103 health records analysed	N/A	Questionnaire and health record review	Descriptive statistics and univariable and multivariable regression	75
Linsky et al^49^ 2015	USA	To identify patient perspectives on intentional discontinuation of prescription medications	N/A	27	Semistructured interviews and focus groups	Modified grounded theory approach	75
Linsky et al^50^ 2015	USA	To understand the attitudes and beliefs of prescribers towards polypharmacy and medication deprescribing	20 prescriber participants 11 GPs 3 NPs 6 pharmacists	N/A	Semistructured interviews	Thematic analysis informed by grounded theory	75
Linsky et al^51^ 2017	USA	To conduct a national survey of primary care providers to identify preferences for interventions to enhance their capability to deprescribe	304 GPs 68 NP or physician assistants 39 CPs	N/A	Questionnaire	Descriptive statistics	75
Linsky et al^52^ 2017	USA	To determine the characteristics of patients who are more or less likely to report discontinuing medications	N/A	803 US Veterans	Questionnaire	Bivariate analysis of association logistic regression	75
Luijks et al^53^ 2012	Netherlands	To explore GPs main aims in the management of multiple morbidity and what influences management in daily practice	25 GPs	N/A	Focus groups	Constant comparative analysis	100
Magin et al^54^ 2015	Australia	Explore GPs' potentially inappropriate prescribing in community dwelling older adults	22 GPs	N/A	Semistructured interviews	Thematic analysis	75
Moen et al^55^ 2009	Sweden	To describe multiple medicine use from the perspective of the older adul.	N/A	59	Focus groups	Content analysis	75
Moen et al^56^ 2010	Sweden	To understand GP's perspective of treating older adult users of multiple medicines	31 GPs	N/A	Focus groups	Content analysis	75
Ng et al^57^ 2017	Singapore	To identify patients accepting of deprescribing and their attitudes to deprescribing	N/A	136	Questionnaire	Descriptive statistical analysis	75
Reeve et al^58^ 2013	Australia	Explore differences in attitudes, beliefs, and previous experiences regarding polypharmacy and stopping current medications	N/A	100	Questionnaire	Descriptive statistical analysis, confidence intervals	75
Reeve et al^59^ 2016	Australia	To explore the beliefs and attitudes of older adults and informal carers toward deprescribing	N/A	14 older adults 14 informal carers	Focus groups	Directed content analysis and conventional content analysis.	100
Riordan et al^60^ 2017	Ireland	To identify the determinants of GP prescribing for older adults and to explore their views on intervention strategies	16 GPs	N/A	Semistructured interviews.	Content analysis using framework approach	100
Schopf et al^61^ 2017	Germany	To identify the perceptions of older adults and GPs regarding communication about polypharmacy and medication safety	1 GP 1 GP trainee 1 medical student	6	Semi‐structured interviews	Content analysis using framework approach	75
Schuling et al^62^ 2012	Netherlands	To explore the feelings of experienced GPs regarding deprescribing and their involvement of older patients in decision making	29 GP participants	N/A	Focus groups	Thematic analysis	75
Sinnige et al^63^ 2016	Netherlands	To explore strategies GPs use when optimising medication regimens for older adults with polypharmacy	12 GPs	N/A	Focus groups	Content analysis	75
Sinnott et al^64^ 2015	Ireland	To explore how and why GPs make decisions when prescribing for multimorbid patients	20 GPs Median age of patient cases selected: 75 y	N/A	Semi structured interviews	Grounded theory approach	75
Sirois et al^65^ 2016	Canada	To describe community‐dwelling older individuals' attitudes and perceptions towards deprescribing	N/A	129	Questionnaire	Descriptive statistics	50
Smith et al^66^ 2010	Ireland	To document the views and beliefs of GPs and pharmacists on managing multiple morbidity in primary care.	13 GPs 7 pharmacists	N/A	Focus groups	Content analysis	75
Sondergaard et al^67^ 2015	Nordic countries	To explore GPs' views and attitudes towards problems and challenges related to the treatment of patients with multimorbidity	180 GPs 76 GPs completed questionnaires	N/A	Recorded workshop discussions and open response questionnaire	Framework analysis	75
Straand and Sandvik^68^ 2001	Norway	To understand the level of patient and prescriber agreement when medication/s have been discontinued	272 GPs	272	Questionnaires paired prescriber and patient	Kappa statistics	75
Turner et al^69^ 2017	Canada	To determine awareness of medication harm and familiarity with the term deprescribing among older community‐living adults.	N/A	2665	Questionnaire	Descriptive statistics and regression analysis	100
Wallis et al^70^ 2017	New Zealand	To explore the barriers and facilitators to deprescribing as reported by primary care physicians in everyday practice	24 GPs	N/A	Semistructured interviews	Multistage coding based on grounded theory	75
Weir et al^71^ 2017	Australia	To explore the reasons behind the variation in patient preferences, attitudes, and experiences in the context of deprescribing	N/A	30 older adults 15 companions	Semistructured interviews	Phenomenological approach	75

Abbreviations: ADEs, adverse drug reactions; CP, consultant pharmacists; EBM, evidence‐based medicine; GPs, general practitioners (this includes primary care family physicians); N/A, not applicable; NP, nurse practitioners; QoL, quality of life

The majority of the studies (n = 28) used qualitative methods, eg, focus groups, semistructured interviews, and Delphi approach; one used mixed methods,^41^ and 9 used quantitative methods.^44^, ^48^, ^51^, ^52^, ^57^, ^58^, ^65^, ^68^, ^69^

### 3.1 Quality of the included studies

The quality of the included studies varied (see Table 1). All but one study^44^ reached 50% or more in the MMAT appraisal score. The 2 researchers who applied the MMAT came to a consensus, prior to the use of the tool, on the meaning of the criteria applied to the qualitative studies. This is recommended by the MMAT developers.^31^ This may mean that we applied the tool differently than others. Few qualitative studies included researcher reflexivity as required by the MMAT. Response rates for the quantitative surveys of GPs were generally low, with no study reaching the required MMAT response rate (≥60%). However, it should be noted that a low response rate is typical when surveying this population^72^ and that the responses were adequate to address the descriptive, exploratory aims of the studies. The 2 studies that used a modified Delphi technique were not scored, as the criteria used in the tool were found to be not applicable.

### 3.2 Findings

Seven key categories were identified that influence deprescribing. These were the health care system, older adult and GP characteristics, knowledge limitations, beliefs about medication use, GPs' perceptions of older adults, older adults' perception of GPs, and fears regarding deprescribing.

#### 3.2.1 The health care system

The structures and practices within the health care systems represented in the 38 included studies generated a variety of factors that influenced both GP prescribers and older adults, and were noticeably similar despite the variety of systems represented. These ranged from macroorganisational factors such as how care is managed and distributed across multiple care providers, down to the management of individual practices, for example, regarding time management and delegation of tasks.

Older adults often need to consult with more than one prescriber in the course of managing multiple morbidities; this may result in an increased risk of poor communication regarding medications between prescribers and confusion about responsibilities.^34^, ^35^, ^38^, ^40^, ^46^, ^50^, ^56^, ^61^, ^62^, ^66^, ^67^ General practitioners view this as a potential barrier to enacting deprescribing. They felt that specialist prescribers regarded their treatment as a priority^62^, ^64^ and focused on a specific area, resulting in no one taking responsibility overall.^66^, ^67^ Also, patients sometimes believed that specialists had more authority than GPs^34^, ^49^, ^59^ and faced confusion about which prescriber was responsible for, and authorised to deprescribe.^49^

Conversely, GPs and older adults valued the long‐term relationships that they developed in the primary care context, and this worked to facilitate deprescribing. Familiarity allowed GPs to build trust and gain an overview of their older patients' preferences, health concerns, and medications.^35^, ^50^, ^53^, ^70^ General practitioners generally felt that this positioned them as the gatekeepers or coordinators of their older patients' care.^53^, ^60^, ^66^, ^67^ However, this perception was contradicted by another recurrent dialogue where GPs noted that the hierarchy between themselves and their specialist counterparts prevented them from questioning medication prescribing decisions, even if it meant continuing medications with no clear indications or that were potentially inappropriate.^35^, ^38^, ^40^, ^46^, ^50^, ^54^, ^56^, ^62^, ^63^

Both older adults and their GPs thought that there was not enough time during consultations to review medications, consider patient preferences, and determine the priorities patients valued.^34^, ^37^, ^38^, ^39^, ^48^, ^50^, ^53^, ^55^, ^56^, ^60^, ^61^, ^63^, ^64^, ^66^, ^70^ Furthermore, GPs were not adequately reimbursed for more complex consultations, especially with patients with multiple morbidities,^53^, ^67^ including undertaking medication reviews, and deprescribing discussions and follow‐up.^34^, ^35^, ^46^, ^56^ Some GPs avoided addressing complex issues because of their awareness of the lack of time.^60^, ^66^

Access to support alongside or within individual practice organisations may influence deprescribing. Changes in practice were suggested, including the ability to consult with pharmacists,^50^, ^51^, ^60^, ^62^, ^63^ inviting fellow GPs to conduct independent prescribing reviews,^38^ phone consults with geriatricians and/or specialists,^66^ and referrals to services able to provide nonpharmacological options.^70^ However, it is notable that the GPs in the study by Sinnige et al^63^ only sought help when their patient's condition did not improve. The use of information technology applications designed to monitor and support prescribing was met with some caution. These were regarded as not well suited to prescribing in the context of the complex needs of the individual older patient with multiple morbidities.^50^, ^60^, ^63^

Delegation of tasks to other team members was mentioned,^51^, ^70^ such as medication reconciliation to nurses^50^ or follow‐up to pharmacists.^57^, ^58^ In practice, this may not happen, as some GPs were uncomfortable with delegation, fearing it would undermine their accountability and authority as a prescriber.^50^

Disease‐specific treatment guideline use generated much concern among GPs, who thought that they promoted ongoing prescribing and encouraged polypharmacy.^35^, ^37^, ^56^, ^62^, ^66^, ^70^ General practitioners noted that guidelines did not take into account treatment decisions in the context of multiple morbidities or provide guidance on the appropriate circumstances in which to discontinue medications.^37^, ^46^, ^47^, ^53^, ^56^, ^62^, ^64^, ^67^ Additionally, GPs felt unsure of the usefulness of guidelines, because they were based on clinical trials that rarely included older adults, especially those with multiple morbidities,^37^, ^46^, ^56^ and did not take into account outcomes valued by the patient.^46^

The way guidelines were used varied among GPs, with those in the study of Sinnott et al^64^ prioritising the management of one disease over another, whereas those in another study^62^ seemed to apply guidelines one after another, without ranking which treatment was most essential. Some GPs were less concerned about following guidelines when prescribing for older patients, preferring to prioritise quality of life,^53^, ^63^ while others thought that the use of guidelines would ensure best practice.^46^ General practitioners were hoping for more useful tools to help them rank the treatment of various diseases,^67^ recognise potentially inappropriate medications,^39^ or guide deprescribing of specific classes of medication.^44^

#### 3.2.2 Older adult and GP characteristics

General practitioners' approaches to the management of their older adults' medications varied.^46^, ^50^, ^63^ A range of characteristics influenced this. General practitioners who had more years of clinical experience were less likely to be concerned about adhering to clinical guidelines, and based their decisions on their clinical reasoning skills and being mindful of the individual needs of their older patients' overall treatment and preferences.^34^, ^56^, ^62^, ^64^ General practitioners who had deprescribed medications successfully in the past were more likely to do so in the future.^34^, ^50^

General practitioners ranked factors that might motivate them to deprescribe, from most to least important and used this as a guide. These factors included cognitive impairment, limited life expectancy, wishes of patient or family, number of medications, and the level of functional dependence.^48^ These changing clinical characteristics encouraged GPs to consider deprescribing because they perceived that the risk of medication continuation exceeded the risk of deprescribing.^48^, ^50^ In this context, some GPs felt more comfortable introducing the topic of deprescribing during discussions with patients about their quality of life versus life expectancy,^62^ although others remained uncomfortable believing their patients would think they had been given up on.^48^, ^62^, ^64^, ^70^

Concerns about polypharmacy use in their older adult patients were frequently raised by health care providers.^34^, ^35^, ^40^, ^50^, ^53^, ^56^, ^62^, ^66^, ^67^ However, their views of what constituted polypharmacy varied,^50^, ^56^ and this was often assessed in an individual, case‐by‐case manner.^34^, ^50^, ^56^ Increased pill burden or the number of medications was a factor in triggering a medication review with a view to deprescribing.^48^ Some prescribers in another study thought that polypharmacy should be framed as a risk to patients who are aging in the same way as they would frame a discussion about the risk of other health problems such as stroke.^34^

Older adults varied in their interest for more information about their medications and involvement in decision making.^61^, ^71^ For example, raising the topic of discontinuing a medication was initiated by only 18% of older adults in a Norwegian study,^68^ while 55% had done so in a US study^51^ and 42% in a Canadian study.^69^ The Canadian study found that initiating a deprescribing conversation was more likely in older adults who searched for information about and had an awareness of medication harms.^69^ In contrast, some older adults felt fearful about expressing their medication preferences and did not share their experience of trialling deprescribing themselves until after the fact.^42^, ^61^, ^71^

#### 3.2.3 Knowledge limitations

Knowledge limitations were commonly cited as a major barrier. General practitioners reported that knowledge gaps, particularly in their pharmacological knowledge, negatively impacted their confidence and willingness to deprescribe.^34^, ^35^, ^50^, ^60^, ^62^, ^66^ They were unsure of the potential interactions between multiple medications,^35^, ^56^, ^62^ unsure of the ongoing benefit of long‐term medication use (eg, preventives),^43^, ^56^, ^62^ and unsure of the outcome of deprescribing.^34^, ^37^ In practice, then, GPs make assessments of the potential harms or benefits of medication based on the individual needs of their patients.^53^, ^54^ Additionally, GPs lacked information about nonpharmacological options^39^ and how to develop, implement, and monitor a deprescribing plan.^44^ Furthermore, GPs in the study by Riordan et al^60^ noted that they were still influenced by pharmaceutical company salespeople, even though they perceive their information to be biased.

An incomplete clinical picture regarding their older patients was another knowledge limitation that negatively impacted GPs' capability to deprescribe. This may result from patients not communicating important information regarding their medications.^35^, ^37^, ^50^, ^56^, ^61^ Furthermore, other prescribers, such as medical specialists, may not effectively communicate all their treatment plans (eg, medication indications and/or duration of treatment) to the GP.^34^, ^35^, ^38^, ^40^, ^46^, ^48^, ^50^, ^51^, ^70^

Older adults had limited knowledge about their medications. The study by Weir et al^71^ found that this affected their confidence to initiate deprescribing discussions. The reasons for and the potential harms of their medications were not always known,^71^ or the ongoing need for some medications was not clear.^55^ Patients assumed, as their doctor had more medical knowledge than they did, that it must be appropriate to continue their medication, otherwise, their doctor would stop writing repeat prescriptions.^59^ Poor health literacy may also be a factor that influences deprescribing decision making.^41^ General practitioners in Clyne et al^39^ earlier study thought that they may be able to negotiate with patients to motivate them to consider deprescribing if they could give their patients more information about their medications.

Previous experiences of deprescribing varied, with 34% in a US study,^51^ 40% in a Canadian study,^65^ and 55% in an Australian study^58^ having experienced deprescribing. Knowledge of the process of deprescribing was limited, for example, with regard to tapering dosages or trialling deprescribing^59^, ^68^ or with regard to the potential reduction in risks that deprescribing offers.^45^

#### 3.2.4 Beliefs about medication use

General practitioners generally held positive beliefs about medications. Their previous experiences of the clinical usefulness of medications and their belief that they generally cause few serious side effects resulted in them favouring continued prescribing over deprescribing.^37^, ^43^ This bias towards prescribing was further promoted by the uncertain outcome of deprescribing.^34^, ^70^

In comparison, older adults hold conflicting ideas around medication use, often expressing concurrent positive and negative beliefs.^36^, ^41^ These conflicting beliefs are clearly illustrated in the 3 quantitative studies that used the Patient's Attitudes Toward Deprescribing survey. Reeve et al,^58^ Sirois et al,^65^ and Ng et al^57^ found that while a majority of older adults thought that all their medications were necessary (95%, 84%, 89%, respectively), similar numbers indicated a willingness to consider deprescribing, if their doctor thought it were possible (92%, 71%, 93%, respectively).

Older adult responses in many of the qualitative studies elaborate on the reasons for their conflicting beliefs. Medications were valued, as they were perceived to extend life and improve well‐being, and their use was to be expected during older age.^71^ Older adults were more likely to believe that their medications were necessary following the testimony of their GP,^59^ recall of the usefulness of medications for family members and friends,^71^ ongoing symptom relief,^36^, ^41^, ^71^ or avoidance of preventable health issues.^55^ Concurrently, older adults expressed a strong dislike of using medications long‐term. They prefer to take as few as possible.^42^, ^49^, ^55^, ^68^, ^71^ Sometimes, nonpharmacological options were followed in order to stop medications.^42^ From a practical point of view, taking multiple medications was perceived to be a burden by some,^36^, ^71^ and notably, in the US health system, was costly.^42^ When taking into account competing outcomes, older adults valued ongoing quality of life more than extending life expectancy, suggesting that, if the side effects from a particular medication were too significant, they may consider discontinuing that medication.^45^, ^59^

#### 3.2.5 GP perceptions of older adults

General practitioner perceptions of their older patients influenced their willingness to consider raising the topic of deprescribing. General practitioners perceived their older patients to be generally resistant to change and that they would be unlikely to accept their advice to deprescribe,^35^, ^37^, ^43^, ^50^, ^62^ especially if they suggested stopping a medication which the older adult perceived was giving them symptom relief.^37^ Alongside this, some believed that older adults themselves had no problem with polypharmacy.^62^ Explanation of potential risks and uncertainties was seen as being particularly hard.^47^, ^64^ General practitioners in the study by Schuling et al^62^ noted that explaining deprescribing to their older patients was made more difficult because of their patients' age and sometimes their poor education. Another study, however, also of Dutch GPs, noted that some believed that even very old people were capable of entering into a shared decision‐making process.^53^

General practitioners felt pressured to meet their older adults' (and/or family members') expectations to prescribe medications,^37^, ^38^, ^39^, ^54^, ^56^, ^60^, ^70^ although some noted that with careful explanation, they may accept the suggestion of alternative treatments.^37^, ^38^, ^39^ General practitioners in the study by Wallis et al^70^ observed that it is important to remember that patients are not coming to an appointment expecting a discussion about deprescribing.

#### 3.2.6 Older adults' perceptions of their GP

Trust was an important factor mentioned in multiple studies. Older adults' hypothetical interest in deprescribing was associated with a higher physician trust score.^57^, ^58^ However, in practice, those who reported higher trust were less likely to have experienced deprescribing.^52^ Older adults reported that their trust in the prescribing practices of their GP was based on the perceived clinical knowledge of their GP, a belief that their GP would make decisions with their best interests in mind, and on the strength of the relationship established between themselves and their GP based on mutual respect, good communication, and knowledge of their preferences.^36^, ^41^, ^49^, ^55^, ^59^, ^61^, ^68^, ^71^ Sometimes, this level of trust meant that they did not ask for important medication information.^61^ Some older adults did qualify their trust, suggesting that they needed to find information for themselves in order to maintain responsibility for their own health.^55^^,^ 49, 71 Others remarked on the paternalistic nature of their relationship with their GP, although they were generally accepting of this, that it was wise not to argue, and preferred to follow their doctor's advice.^41^, ^61^, ^71^ Weir et al^71^ compared attitudes of older adults across 3 groups and noted that those who were frailer and/or lacked an understanding of their medications were happy to abdicate decision making about their medications, including deprescribing, to their doctor.

Both GPs and older adults recognised that trust could be undermined when different prescribers gave conflicting advice about deprescribing.^38^, ^49^, ^55^, ^62^, ^68^ Finally, others, in the study by Moen et al,^55^ noted a general distrust of the health system rather than of a specific prescriber and were sceptical about the ongoing influence of pharmaceutical companies on prescribers.

#### 3.2.7 Fears

Both older adults and GPs feared the potential for unfavourable outcomes from deprescribing, such as a return of symptoms, withdrawal effects, or previously avoided serious events such as stroke, occurring following the cessation of preventative medications.^34^, ^37^, ^42^, ^50^, ^61^, ^62^, ^68^, ^70^, ^71^ This fear often outweighed the fear of risks associated with continuing multiple, sometimes potentially inappropriate, medications from the perspective of both patients^71^ and prescribers.^37^, ^64^ As a result, maintaining the status quo was preferred. However, older adults' fears of, or experience with, side effects,^42^, ^55^, ^68^ drug interactions when using multiple medications, and/or fear of addiction,^55^ were factors that could influence them to consider the acceptability of deprescribing.

General practitioners feared that a poor outcome as a result of their deprescribing advice would undermine their relationship with their patient or family members^62^, ^64^, ^70^ and could lead to litigation.^61^, ^62^, ^64^ Others sought to shift the responsibility for a recommendation to deprescribe, by referring to the requirements of external parties such as drivers' licensing bodies.^37^

## 4 DISCUSSION

As far as we are aware, this is the first mixed studies review to consider the factors that influence deprescribing for both community‐living older adults and GPs. Compared to earlier reviews, the inclusion of recent quantitative papers allowed us to note the strong interest of older adults in deprescribing if recommended to do so by their doctor. This suggests that GPs' fear of patient resistance may often be unfounded. Additionally, we noted the priority GPs give to various patient related factors when considering deprescribing, including cognitive impairment, limited life expectancy, and patient or family preferences. Knowing the original medication indication, having assistance to monitor deprescribing, and further involvement of patients in shared decision making were ranked as being the most helpful for prescribers to enable deprescribing.

Additionally, we found that the factors that influence deprescribing have remained unchanged over the time period covered by the review and are similar across all of the health systems represented. This is important to note, as there has been an increase in research in this area in the last decade and this appears to have had little impact on primary care practice. In their systematic review of the knowledge to practice gap, Lau et al^73^ argue the importance of moving beyond the identification of barriers and facilitators, if change is to be achieved. Most studies continue to identify barriers and facilitators to deprescribing and examine these as separate factors, without considering the relative importance of each of them or how they interact.

This review of the current literature suggests that the factors that influence deprescribing mostly act as barriers. Some are created by the health system, which provides the context for deprescribing, while other barriers occur at the practice and individual level. At the individual level, Bokhof and Junius‐Walker's^24^ review found that both older adults and GPs faced uncertainty in deprescribing decision making, and this finding is confirmed by this review. Individual level barriers to deprescribing may be grouped into 2 key areas: knowledge limitations and communication gaps. Reducing uncertainty in these 2 areas, together with addressing health system constraints, could lead to change in deprescribing practice.

4.1

#### 4.1.1. Health system constraints

Health system constraints, which can only be addressed at the policy level, provide the context for any change at the practice level. Primary care consultations continue to be best suited to managing acute health problems rather than chronic health conditions or multiple morbidities.^74^ Consultation times remain short, and reimbursement for more complex tasks is still inadequate. These practical issues may mean GPs avoid time‐consuming areas such as discussions about deprescribing, as they seek to meet the needs of all their patients. As a result, deprescribing tends to occur reactively when a significant medication related problem arises or when there are other clear indications such as increasing cognitive impairment or limited life expectancy. However, discussing deprescribing earlier as a routine part of medication management would provide GPs with the opportunity to work collaboratively with the patient, and other members of the health care team, to ensure that medication regimens remain beneficial and manageable for the patient. Several different models of integrated care, which focus on the establishment of multidisciplinary teams or provider networks within primary care settings, have been shown to be successful in improving care for those with chronic diseases.^74^ Such models could also facilitate deprescribing and should be further researched.

#### 4.1.2. Knowledge limitations

General practitioners noted that they lacked pharmacological knowledge in the context of treating older, multimorbid adults. This suggests that medical curricula should be revised to include more specific geriatric pharmacology and deprescribing education,^75^ together with ongoing professional development after graduation. Pharmacists, located either in the community or within GP practices, can also supply information. Those located within GP practices may be better placed to collaborate and meet the immediate information needs of GPs.^76^

The review found that the evidence available to guide GPs when making prescribing decisions is limited. Clinical trials rarely include older adults with multiple morbidities, and as a result, GPs have little evidence of the effect of medications in real‐life patients.^77^ Furthermore, this group is excluded from most randomised control trials for the development of evidence‐based medicine and clinical practice guidelines.^78^ Experienced GPs manage this lack of evidence by drawing on their own clinical knowledge base, deviating from clinical guidelines as required, and taking into account quality of life considerations and patient preferences. This suggests that those who are less experienced may benefit from more structured opportunities to draw on the knowledge base of others such as pharmacists, geriatricians, and peers, to review deprescribing decisions in the context of complex medication regimens.

Paternalistic relationships and the asymmetry of medical knowledge undermine some older adults' confidence to discuss their preferences with their GPs and/or ask questions to clarify information gaps. This finding is similar to an earlier review on communication about medicines between patients and health care professionals.^79^ To ensure that older adults have an opportunity to make informed decisions, changes in medication information for patients are required to ensure that this is accessible and understandable, irrespective of the health literacy level of the individual. This should include information regarding how medications are deprescribed. The lack of awareness of monitored deprescribing as an option is demonstrated by those older adults who discontinued medications themselves, without discussing their decision with their GP.

#### 4.1.3. Communication gaps

The primary care model allows both the patient and their GP to develop a relationship over time and was favoured by both parties in the included studies. This was seen as especially important in the context of managing chronic multiple morbidities. Awareness of patient preferences and open, shared communication in the context of trust reduces uncertainty. The earlier reviews by Reeve et al^25^and Bokhof and Junius‐Walker^24^ identified trust as being an important characteristic that was necessary to facilitate deprescribing, but the role of trust may be more complex than this suggests. In some studies, trust is associated with a willingness to consider deprescribing, suggesting that it may facilitate acceptance of a prescriber's recommendation to deprescribe, even in the face of uncertainty.^57^, ^58^ However, other research demonstrates that trust can also act as a barrier to deprescribing. For example, trust was used by patients to explain why they unquestioningly accepted their medication regimen, resulting in lower reports of deprescribing.^52^ These contradictory actions of trust were also demonstrated in the small study by Schöpf et al^61^ included in this review and are similar to the findings regarding trust in the study by Belcher et al^80^ of older adults' participation in medication related decision making.

Because older adults often have more than one prescriber involved in their care, good communication structures and practices within and between health care providers are especially important. Currently, GPs may receive little information about specialist consultation outcome/s. Furthermore, GPs sometimes continued medications they considered inappropriate because they were not willing to challenge their specialist counterparts, and some older adults believed that only their specialists had the authority to deprescribe. Despite these challenges, GPs remain well placed to act as the overall coordinators of their older adults' medication regimens as they may be the only prescriber who can see the full picture of what is being taken. This suggests that a collaborative approach with improved communication between specialist and GP prescribers is needed, along with clarification of lines of responsibility.

### 4.2 Areas for further investigation

Future deprescribing research needs to explore aspects of diversity among older adults. Age was used to define the samples in the reviewed studies; however, chronological age is only loosely related to physiological changes that occur during the aging process, suggesting that this group should not be thought of as homogenous. The majority of older adults continue to live in the community and are a diverse group that contribute to society as mentors, consumers, members of the workforce, caregivers, and innovators, despite the challenges of the aging process.^17^

The review highlights that several other areas also require further exploration. Work is required to understand the extent to which health literacy, which is known to be lower in this age group,^81^ and socio‐economic characteristics influence older adults' decisions. Additionally, research is required to further understand the complex role of trust within long‐term doctor‐patient relationships, as it applies to deprescribing.

### 4.3 Limitations

Our review only included studies available in English. Despite the search methodology including multiple databases and a variety of relevant search terms, a significant number of the included studies were identified via hand searches of reference lists of related articles and using citation tracking. This reduces the reproducibility of our methods. Finally, the MMAT used to assess the quality of the included studies is still in development, so the quality scores given should be treated cautiously.

## 5 CONCLUSION

This review investigated the factors that influence deprescribing from the perspective of both GPs and older adults aged 65 years or older and living independently in the community. The review found that these factors mostly act as barriers to prevent deprescribing from entering into discussions during consultations. They have remained static across the review period and are similar across health systems. To achieve change, multilevel strategies should be prioritised to address structural constraints within health systems and to manage uncertainty at the practice and individual level, reducing knowledge limitations and closing communication gaps.

## CONFLICT OF INTEREST

The authors declare that they have no conflicts of interest.

## AUTHOR CONTRIBUTIONS

Conceptualization: Robyn Gillespie, Lindsey Harrison, Judy Mullan

Data curation: Robyn Gillespie

Formal analysis: Robyn Gillespie, Lindsey Harrison, Judy Mullan

Writing—original draft preparation: Robyn Gillespie

Writing—review and editing: Robyn Gillespie, Lindsey Harrison, and Judy Mullan

## Supporting information

Appendix S1. Supporting information
