# Supplementary material for: Deprescribing medications for older adults in the primary care context: A mixed studies review
Source: Health Sci Rep. 2018 May 10;1(7):e45. doi: 10.1002/hsr2.45 (PMC6266366; doi:10.1002/hsr2.45)
Supplement: Supplementary file 1 — Appendix S1. Supporting information [file HSR2-1-e45-s001.docx]

**CINAHL**

**"( Physician or "family physician" or "general pract*" or GP or doctor or clinician or prescriber or "health professional" or "health care professional" OR "health personnel" or "health practitioner" ) AND ( ("prescription drug" OR prescribing OR medicines OR medication OR polypharmacy ) ) AND ( ( Withdraw or withdrawing or withdrawal or cease or ceasing or cessation or stop or stopping or discontinue or discontinuing or discontinuation or reduce or reducing or reduction or deprescribe or deprescribing or optim* ) )**

**Limiters** - Full Text; Published Date: 20000101-20171231

**Expanders** - Apply equivalent subjects

**Narrow by SubjectAge:**- aged: 65+ years

**Narrow by Language:**- English

**Search modes** - Find all my search terms

Results 432
